# Alligators as West Nile Virus Amplifiers

**DOI:** 10.3201/eid1012.040264

**Published:** 2004-12

**Authors:** Kaci Klenk, Jamie Snow, Katrina Morgan, Richard Bowen, Michael Stephens, Falicia Foster, Paul Gordy, Susan Beckett, Nicholas Komar, Duane Gubler, Michel Bunning

**Affiliations:** * Centers for Disease Control and Prevention, Fort Collins, Colorado, USA;; † Colorado State University, Fort Collins, Colorado, USA;; ‡ United States Air Force, Washington, DC, USA

**Keywords:** West Nile virus, flavivirus, arbovirus, alligators, reptiles, transmission, viremia, research

## Abstract

Recent evidence suggests that American alligators (*Alligator mississippiensis*) may be capable of transmitting West Nile virus (WNV) to other alligators. We experimentally exposed 24 juvenile alligators to WNV parenterally or orally. All became infected, and all but three sustained viremia titers >5.0 log_10_ PFU/mL (a threshold considered infectious for *Culex quinquefasciatus* mosquitoes) for 1 to 8 days. Noninoculated tankmates also became infected. The viremia profiles and multiple routes of infection suggest alligators may play an important role in WNV transmission in areas with high population densities of juvenile alligators.

## Abstract 2

Juvenile alligators may help transmit West Nile virus in some areas.

The primary enzootic cycle for West Nile virus (WNV) is between adult ornithophilic mosquitoes and birds, with these mosquitoes occasionally infecting incidental hosts such as horses and humans (*1*). Most research to date has focused on these endothermic vertebrate hosts. Other arboviruses infect a variety of ectotherms, including species of lizards (*2**–**4*), snakes (*5**–**11*), and turtles (*12**,**13*), but the knowledge of ectotherm involvement in the ecology of WNV is limited. In the lake frog (*Rana ridibunda*), West Nile viremia capable of infecting mosquitoes (*14**,**15*) develops, and antibodies develop in Nile crocodiles (*Crocodylus niloticus*) and other ectotherms after natural infection (*16**,**17*). Experimentally infected North American bullfrogs (*R. catesbeiana*) and green iguanas (*Iguana iguana*) sustain low viremia levels for a short period of time, which suggests that they do not transmit the virus to biting mosquitoes (*18*).

In North America, WNV infections in ectotherms were first reported in 2001 (*19*). In the years 2001 to 2003, U.S. alligator farms reported substantial economic losses and at least one human case of fever due to WNV outbreaks in juvenile American alligators (*Alligator mississippiensis*) (*19**,**20*; L. Tengelsen, pers. comm.). These alligators were housed in crowded tanks at a constant temperature of 32°C. The mode of transmission, the risk posed to handlers, and role of alligators in secondary WNV transmission cycles are unknown. To assess the potential role of juvenile alligators in the ecology of WNV transmission, we evaluated routes of transmission, determined viremia profiles, evaluated viral persistence in organs, and examined the role of temperature on WNV replication in these animals.

## Materials and Methods

### Acquiring and Housing Alligators

American alligators were transported to Fort Collins, Colorado, from two U.S. alligator farms: St. Augustine Alligator Farm, St. Augustine, Florida (N = 26, age = 1–2 years, weight = 1–3 kg) and Colorado Gator Farm, Mosca, Colorado (N = 22, age = 10 mo, weight = 200–400 g). Alligators were fed gator chow pellets (Burris Mill and Feed, Franklinton, LA) twice per week (food volume ≈5% of body weight) (*20*).

Alligators were divided between two rooms; one room was maintained at 32°C and the other at 27°C. Room temperature and humidity were monitored by HOBO data recorders (Onset, Bourne, MA). Within each room, alligators were placed in livestock tanks (2 m diameter) separated by plastic curtains to reduce cross-contamination between tanks. Each tank contained 15 cm of water at the corresponding temperature (27°C or 32°C) and an adequate basking surface. Water was heated with aquarium heaters and aerated with an aquarium water pump. Equipment was checked twice daily, and the water was changed and tanks were disinfected every other day. Rooms were kept dark to calm the alligators (a standard practice at some alligator farms).

### Mouse Infection

The NY99-4132 strain of WNV, passaged 3–4 times in Vero cells, originally from crow brain provided by W. Stone, New York State Department of Environmental Conservation, Albany, New York, was used in this study. We injected 24 Swiss Webster mice (6–8 weeks of age) subcutaneously with ≈1,000–2,000 PFU of WNV. Mice that developed neurologic signs 7–8 days postinoculation were euthanized and frozen at –70°C.

### Alligator Infection

Six alligators in the 32°C room and six alligators in the 27°C room were subcutaneously injected behind the left front leg with ≈7,500 PFU of WNV with a volume of 0.15 mL. Another six animals from each room were fed WNV-infected mice (1/2 mouse per small alligator [<700 g] and 1 mouse per larger alligator [>700 g]). Two noninfected alligators were placed with each infected group to serve as tankmate controls. Eight noninfected alligators served as bleeding controls in each room.

### WNV Isolation from Serum

Blood samples were collected from each alligator daily for 15 days postinfection for virus isolation (some tankmate alligators were bled daily through day 21). Blood (0.2 mL) was collected from the caudal vein and added to 0.9 mL of BA-1 diluent (composed of Hank's M-199 salts, 1% bovine serum albumin, 350 mg/L sodium bicarbonate, 100 U/mL penicillin, 100 mg/L streptomycin, 1 mg/L amphotericin B in 0.05 mol/L Tris, pH 7.6), producing an approximate 1:10 serum dilution. Blood samples were centrifuged at 3,750 rpm for 10 min to separate serum from clotted blood and stored at –70°C.

WNV viremia was quantified by plaque assay. Blood samples were serially diluted 10-fold with BA-1 through 10^–8^, and 100 mL of each dilution was added in duplicate to Vero cell monolayers in six-well plates (Costar, Cambridge, MA). Samples were allowed to incubate on the cells for 1 h at 37°C. Cells were then overlaid with 3 mL per well of 0.5% agarose in M-199 medium, supplemented with 350 mg/L sodium bicarbonate, 29.2 mg/L L-glutamine, and antimicrobial drugs as in BA-1. After 48 h of incubation, a second 3-mL 0.5% agarose overlay containing 0.004% neutral red was added for plaque visualization. Plaques were counted on day 4 postinfection.

### WNV Isolation from Other Samples

Cloacal swab samples were taken from each alligator daily for 15 days postinfection (some tankmate alligators were swabbed daily through day 21 postinfection). A cotton swab was inserted into the cloaca ≈2 cm, rotated, and then placed in a tube containing 1.0 mL BA-1. Virus content was quantified by plaque assay.

Nine alligators (two that died of infection and seven that recovered) were tested for virus in tissues. Tissue samples (≈0.5 cm^3^ in size) were harvested from the lung, liver, spleen, heart, kidney, spinal cord, cerebrum, and cerebellum. Samples were trimmed as needed and ground in 1.5 mL BA-1 containing 20% fetal bovine serum with a Retsch MM300 mixer mill (Retsch GmbH & Co, Hann, Germany) (30 cycles/sec for 4 min). Each resulting homogenate was transferred to a 1.7-mL Eppendorf microcentrifuge tube and clarified by microcentrifugation at 7,500 rpm for 3 min. Each supernatant was transferred to a 1.8-mL cryovial (Nalge Nunc International, Rochester, NY) and stored at –70°C. Virus content was quantified by plaque assay.

Water (0.5 mL) was taken from each tank daily (before cleaning) for 15 days postinfection and then twice per week through day 31 postinfection. Water samples were added to 0.5 mL BA-1 (containing 2x concentrations of antimicrobial drugs). Water samples were pooled according to tank. Half of each pool was used for virus isolation. Water samples were added to 25-cm^3^ tissue culture flasks (Corning, Corning, NY) (1 mL per flask) containing Vero cell monolayers. Flasks were rocked every 15 min for 1 h at 37°C, and 10 mL of Dulbecco's Modified Eagle Medium (Invitrogen, Carlsbad, CA), supplemented with 2% fetal bovine serum, was added to each flask. Flask media were replaced on day 6 postinfection. Flasks were checked daily for cytopathic effect (CPE) through day 10 postinfection. Remaining water samples were tested by Taqman reverse transcriptase–polymerase chain reaction (RT-PCR) (*21*).

### Neutralizing Antibody Detection

Blood samples (0.4–0.6 mL) were collected from each alligator for neutralizing antibody detection twice per week from day 21 postinfection through day 31 postinfection. To detect neutralizing antibodies, 15-μL serum samples from day 21 to day 31 were mixed with 60 μL of BA-1 and 75 μL of a WNV preparation (200 PFU/0.1 mL) in a polypropylene 96-well plate (Costar, Cambridge, MA). The virus-serum mixtures were incubated at 37°C for 1 h to allow for virus neutralization. These mixtures were then tested by plaque assay. Controls employed BA-1 only (cell viability control), serum-free virus mixture with BA-1 only (to enumerate PFU in the challenge dose of virus), and West Nile hyperimmune mouse ascitic fluid (diluted 1:200) mixture with virus (to verify challenge virus identity). Specimens were considered positive for WNV neutralizing antibodies if they reduced a challenge dose of ≈100 PFU of WNV by at least 90% at a serum dilution of 1:10.

## Results

### Viremia after Parenteral Infection

Every alligator injected with WNV became viremic from days 1 to 3 postinfection (Figure A and B). Alligators housed at 32°C became viremic on day 1 or 2 postinfection, while those kept at 27°C became viremic on days 2 or 3 postinfection. Viremia in the 32°C alligators persisted an average of 10 days with an average maximum WNV titer of 5.7 log_10_ PFU/mL (maximum 6.7 log_10_ PFU/mL). The alligators housed in 27°C conditions were viremic for ≈14 days and averaged a maximum WNV titer of 5.8 log_10_ PFU/mL (maximum 6.1 log_10_ PFU/mL). No injected alligators died of the infection.

**Figure:**
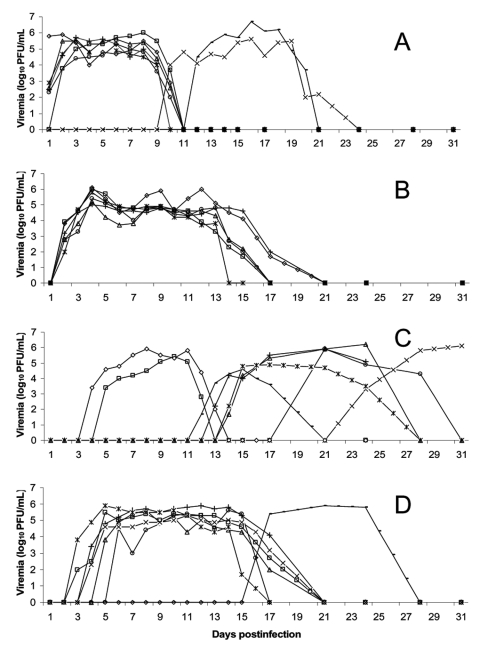
Daily viremia titers. A) Injected alligators held at 32°C (◊, *, ○, Δ, □, +) and their tankmates (x, ◊), B) Injected alligators held at 27°C (◊, ○, *, Δ, □, +). Tankmates did not become viremic. C) Orally infected alligators held at 32°F (◊, □) and their tankmates (○, ◊, *, +, x, Δ). D) Orally infected alligators held at 27°C (◊, ○, *, Δ, □, +) and their tankmates (x, ◊). Blood samples were collected from each alligator for virus isolation once a day for 15 days postinfection. (Some tankmate alligators were bled daily through day 21 postinfection.) After day 15, alligators were bled biweekly through day 31 postinfection. West Nile viremia was quantified by using a Vero cell plaque assay. Plaques were counted after 4 days of incubation. The threshold of detection was 1.7 log_10_ PFU/mL of serum. Values <10^1.7^ were considered to be zero.

Tankmates in the 32°C injected group became viremic on days 10 and 12 postinfection, while the tankmates in the 27°C injected group failed to become viremic (Figure A and B). Infection of tankmates in the 32°C injected group persisted for ≈10 to 12 days, and neither died of the infection.

### Viremia after Oral Infection

Viremia developed in two alligators from the 32°C room and five alligators from the 27°C room 3–6 days after they ate WNV infected mice (Figure C and D). Alligators in the 32°C room remained viremic for >9 days, while the alligators in the 27°C room remained viremic for ≈14 days.

Every alligator in the 32°C orally infected tank eventually became viremic during the experiment, with an average maximum WNV titer of 5.6 log_10_ PFU/mL (max 6.2 log_10_ PFU/mL) (Figure C). Tankmate viremia onset ranged from 12 to 24 days after infection. Because we stopped routine daily bleeding after day 15 postinfection, the exact viremia onset days of two alligators in this group are unknown. Also, the average duration of viremia for these alligators cannot be calculated. Two alligators in this group died of WNV infection after 12 or 13 days of viremia.

Both tankmates from the 27°C orally infected group also became infected (Figure D). One tankmate came into contact with a viremic mouse but did not eat it; this alligator became viremic on day 4 postinfection, and the infection persisted for >14 days. Viremia developed in the other tankmate on day 16 postinfection. Because of the absence of daily bleeding, the duration of viremia is not precisely known.

### Viral Loads of Cloacal Swabs

Of 29 viremic alligators, 24 had detectable viral loads in their cloacae (Table 1). All five remaining infected alligators became viremic on the last 1 to 2 days of swabbing or after daily swabbing ceased, so no positive swabs can be reported from them. Viral shedding was detected within 3 days of detectable viremia and, in some instances, was detected on the same day as viremia onset. Duration of shedding lasted 6 to >12 days, with an average maximum viral load of 5.2 log_10_ PFU/swab (maximum 6.2 log_10_ PFU/swab).

**Table 1:** West Nile virus isolation from cloacal swabs of infected alligators^a^

Tank	Status	No. with WNV-positive swabs	Mean first day viral shedding^b^	Mean duration viral shedding (d)	Mean maximum viral load and range (log_10_ PFU/swab)
32°C parenteral	Infected (n = 6)	6	2	≥12	4.4 (3.5–4.9)
	Tankmate (n = 2)	2	12	≥9	5.9 (4.9–6.2)
32°C oral	Infected (n = 2)	2	6	≥8	4.9 (3.3–5.2)
	Tankmate (n = 6)^c^	4*	15	≥3	4.3 (2.0–4.8)
27°C parenteral	Infected (n = 6)	6	2	≥9	4.0 (1.9–4.4)
	Tankmate (n = 2)	0	NA	NA	NA
27°C oral	Infected (n = 6)	5	6	≥10	4.2 (1.9–4.7)
	Tankmate (n = 2)	1*	7	≥9	2.6 (NA)

^a^For some alligators (*), daily swabbing had stopped before or immediately after infection, so positive cloacal swabs were not detected. ^b^Days after injection or oral infection of the alligators; NA, not applicable. ^c^Four of six alligators were fed WNV-infected mice, but most likely became infected by tankmate transmission rather than oral transmission.

### Viral Isolation from Other Samples

Of 29 infected alligators, 2 died, and WNV was detected in their tissues (Table 2). No virus was isolated from the seven alligators that recovered from infection. WNV neutralizing antibodies were detected in 100% of infected alligators within 25 days after virus detection. No infectious virus or viral RNA was detected in water samples. Sample volumes were each 0.00013% of the total tank water volume.

**Table 2:** West Nile virus isolation from tissues of the two alligators that died^a^

Alligator	Tank	Day after viremia onset	Tissue (log_10_ PFU/0.5 cm^3^)							
			Heart	Kidney	Spleen	Liver	Lung	Spinal cord	Cerebellum	Cerebrum
M0216	32°C oral tankmate	12	5.8	<0.9	<0.9	1.4	6.1	2.1	2.7	1.6
M0228	32°C oral tankmate	15	<0.9	2.2	2.5	1.6	3.5	NA	<0.9	<0.9

^a^No virus was detected in tissues from seven recovered alligators tested.

## Discussion

In some southern states, alligator farms contribute to the economy as agricultural producers and tourist attractions. A typical operation raises 3,000 alligators each year. The market value of raw products (e.g., meat, hides) from an average adult alligator is ≈$300, and alligator meat typically fetches ≈$5 per pound. In Louisiana alone, the total value of farm-raised alligators is >$16 million (*22*). Beginning in 2001, alligator farms in at least four different states suffered substantial economic losses due to WNV outbreaks in young alligators. Public health risks involved in these large outbreaks and the eventual culling of thousands of young alligators are also substantial.

We have shown that sick juvenile alligators carry high viral loads in tissues, which poses a threat to handlers, processors, and consumers, although this risk has not been quantified beyond one reported case in Idaho of human West Nile fever in a handler of imported Florida juvenile alligators. Furthermore, all infected alligators in our study shed WNV from the cloaca, which poses another possible threat to other alligators and to handlers. Although tankmates in our study became infected at a high rate, we cannot conclude with certainty that cloacal shedding is the cause of this direct transmission.

Direct transmission likely plays an important role in the epizootiology of WNV infection in farmed alligators but has not been documented in wild alligators (*19**,**20*). However, we now know that high levels of viremia develop in young alligators, so WNV infection could likely lead to mosquitoborne transmission as well. In general, viremia reached titers considered to be infectious to *Culex quinquefasciatus* mosquitoes with the NY99 strain of WNV (5.0 log_10_ PFU/mL) in all but three infected alligators (*23**,**24*). *Cx. quinquefasciatus* is one of the principal vectors of WNV in the southeastern United States (*25*). Numerous species of mosquitoes feed on reptiles as well as birds and mammals and thus could be vectors from alligators to people (*26*). The primary WNV amplification cycle is believed to depend on birds and mosquitoes (*1*); however, the maximum duration of viremia in juvenile alligators was >2 weeks, which is longer than that observed in birds (maximum duration 7 days) (*27*).

Because most alligator farms raise juvenile alligators at a higher temperature (32°C) than older alligators, the effect of temperature on WNV infection was of interest. The 5°C difference in temperature that we tested did not significantly alter infection rates (Fisher exact test, p = 0.11). In general, alligators housed at 27°C maintained detectable viremia 4–5 days longer than the alligators housed at 32°C, which could be due to an enhanced immune function at the higher temperature. In 1969, Tait et al. discovered that lizards (*Egernia cunninghami*) housed at 30°C produced higher titers of antibodies at a faster rate than those housed at 25°C after injection with sheep red blood cells (*28*). In our study, WNV neutralizing antibodies developed in all infected alligators within a month of infection; these antibodies were detected in the alligators housed at 32°C an average of 5 days earlier than in the alligators housed at 27°C (data not shown). Although neutralizing antibody circulation is only one part of immune function, previous studies have suggested that multiple aspects of the ectothermic immune system may be affected by body temperature, which is directly affected by environmental temperature (*29**–**31*).

Transmission of WNV by means other than mosquitoes has been shown in humans (*32**–**34*), mice (*35*), and birds (*27**,**36*), although some modes of transmission are poorly understood. In our study, alligators were successfully infected by parenteral and oral routes, although infection rates between the parenteral and oral groups differed significantly (Fisher exact test, p < 0.05). All 12 injected and 7 of 12 orally inoculated alligators became viremic. Furthermore, high viral loads in the cloacal samples indicate a possible fecal-oral route of transmission, although no viral RNA was detected in our water samples, probably because of the dilution effect of ≈400 L per tank (a 10^–6^ dilution factor). Other transmission routes could include bloodborne transmission, although wounds were observed on only two alligators during the experiment, or direct transmission by contaminated water droplets sprayed onto the conjunctiva or other mucous membranes. Although we apparently sampled water that was too dilute to detect WNV particles, at discrete moments, pockets of highly concentrated virus particles in the water could exist and lead to transmission. Infectious saliva could also contribute to direct transmission, but this factor was not examined in this study.

The only deaths observed in our study were two alligators housed at 32°C and infected by tankmate transmission. These data confirm the observations on the farms that WNV infection kills some alligators. Precise death rates on the affected farms are unknown, but we observed an overall death rate of 7% in this study (2 of 29 infected alligators)^2^. Because of infectious virus in their tissues, these dead alligators represent a potential health threat to handlers, alligator meat consumers, and other alligators. Infectious virus was not isolated from tissues of seven alligators that recovered from infection, which suggests that surviving alligators do not pose a health threat after viremia and cloacal shedding cease (within 4 weeks postinfection).

In summary, juvenile alligators may be competent hosts for WNV. This study showed that juvenile alligators have adequate viremia levels (high-titer and long-lasting) for viral transmission by mosquitoes. Coupled with multiple routes of infection, alligators may play a role in WNV ecology, especially in areas where the density of young alligators is high.

## Footnotes

2 Of alligators infected by tankmate transmission, the death rate is 20% (2/10). Of alligators held at 32°C, the death rate is 13% (2/16).

## References

[1] Komar N. West Nile virus: epidemiology and ecology in North America. Adv Virus Res. 2003;61:185–234. 10.1016/S0065-3527(03)61005-5 14714433

[2] Doi R, Oya A, Shirasaka A, Yabe S, Sasa M. Studies on Japanese encephalitis virus infection of reptiles. II. Roles of lizards on hibernation of Japanese encephalitis virus. Jpn J Exp Med. 1983;53:125–34. 6141311

[3] Oya A, Doi R, Shirasaka A, Yabe S, Sasa M. Studies on Japanese encephalitis virus infection of reptiles. I. Experimental infection of snakes and lizards. Jpn J Exp Med. 1983;53:117–23. 6141310

[4] Doi R, Oya A, Telford SR Jr. A preliminary report on infection of the lizard, *Takydromus tachydromoides*, with Japanese encephalitis virus. Jpn J Med Sci Biol. 1968;21:205–7. 4301955 10.7883/yoken1952.21.205

[5] Shortridge KF, Ng MH, Oya A, Kobayashi M, Munro R, Wong F, Arbovirus infections in reptiles: immunological evidence for a high incidence of Japanese encephalitis virus in the cobra, *Naja naja.* Trans R Soc Trop Med Hyg. 1974;68:454–60. 10.1016/0035-9203(74)90068-6 4460310

[6] Mifune K, Shichijo A, Ueda Y, Suenaga O, Miyagi L. Low susceptibility of common snakes in Japan to Japanese encephalitis virus. Trop Med. 1969;11:27–32.

[7] Lee HW. Multiplication and antibody formation of Japanese encephalitis virus in snakes. II. Proliferation of the virus. Seoul J Med. 1968;9:157–61.

[8] Thomas LA, Eklund CM, Rush WA. Susceptibility of garter snakes (*Thamnophis* spp.) to western equine encephalomyelitis virus. Proc Soc Exp Biol Med. 1958;99:698–700. 13614471 10.3181/00379727-99-24468

[9] Thomas LA, Eklund CM. Overwintering of western equine encephalomyelitis virus in experimentally infected garter snakes and transmission to mosquitoes. Proc Soc Exp Biol Med. 1960;105:52–5. 13776510 10.3181/00379727-105-26006

[10] Thomas LA, Eklund CM. Overwintering of western equine encephalomyelitis virus in garter snakes experimentally infected by *Culex tarsalis.* Proc Soc Exp Biol Med. 1962;109:421–4. 13920821 10.3181/00379727-109-27225

[11] Thomas L, Patzer E, Cory J, Coe J. Antibody development in garter snakes (*Thamnophis* spp.) experimentally infected with Western equine encephalitis. Am J Trop Med Hyg. 1980;29:112–7. 7352618 10.4269/ajtmh.1980.29.112

[12] Shortridge KF, Oya A, Kobayashi M, Yip DY. Arbovirus infections in reptiles: Studies on the presence of Japanese encephalitis virus antibody in the plasma of the turtle, *Trionyx sinensis.* Southeast Asian J Trop Med Public Health. 1975;6:161–9. 170690

[13] Shortridge KF, Oya A, Kobayashi M, Duggan R. Japanese encephalitis virus antibody in cold-blooded animals. Trans R Soc Trop Med Hyg. 1977;71:261–2. 10.1016/0035-9203(77)90022-0 888172

[14] Kostyukov MA, Alekseev AN, Bul'chev VP, Gordeeva ZE. Experimentally proven infection of *Culex pipiens* L. mosquitoes with West Nile fever virus via the Lake Pallas *Rana ridibunda* frog and its transmission via bites. Med Parazitol (Mosk). 1986;6:76–8. 3029556

[15] Kostyukov MA, Gordeeva EE, Bulychev VP, Hemova NV, Daniyarov OA, Tuktaev TM. The lake frog (*Rana ridibunda*)—one of the food hosts of blood-sucking mosquitoes in Tadzhikistan—a reservoir of the West Nile fever virus. Med Parazitol (Mosk). 1985;3:49–50. 2863744

[16] Steinman A, Banet-Noach C, Tal S, Levi O, Simanov L, Perk M, West Nile virus infection of crocodiles [letter]. Emerg Infect Dis. 2003;9:887–9. 12899140 10.3201/eid0907.020816 PMC3023443

[17] Nir Y, Lasowski Y, Avivi A, Cgoldwasser R. Survey for antibodies to arboviruses in the serum of various animals in Israel during 1965–1966. Am J Trop Med Hyg. 1969;18:416–22. 4889830 10.4269/ajtmh.1969.18.416

[18] Klenk K, Komar N. Poor replication of West Nile virus (New York 1999 strain) in three reptilian and one amphibian species. Am J Trop Med Hyg. 2003;69:260–2. 14628941

[19] Miller DL, Michael MJ, Baldwin C, Burtle G, Ingram D, Hines ME, West Nile virus in farmed alligators. Emerg Infect Dis. 2003;9:794–9. 12890319 10.3201/eid0907.030085 PMC3023431

[20] Jacobson ER, Ginn PE, Troutman JM, Farina L, Stark L, Klenk K, West Nile virus infection in farmed American alligators (Alligator mississippiensis) in Florida. J Wildl Dis. 2005. In press. 15827215 10.7589/0090-3558-41.1.96

[21] Lanciotti RS, Kerst AJ, Nasci RS, Godsey MS, Mitchell CJ, Savage HM, Rapid detection of West Nile virus from human clinical specimens, field-collected mosquitoes, and avian samples by a TaqMan reverse transcriptase PCR assay. J Clin Microbiol. 2000;38:4066–71. 11060069 10.1128/jcm.38.11.4066-4071.2000 PMC87542

[22] Ohio Pork Industry Center. An engineer eyes hog carcasses as alligator feed [newsletter article on the Internet]. Columbus (OH): The Ohio State University Extension; 2003 Feb [cited 2004 Oct 19]. Available from http://porkinfo.osu.edu/news.archives.html

[23] Turell MJ, O'Guinn M, Oliver J. Potential for New York mosquitoes to transmit West Nile virus. Am J Trop Med Hyg. 2000;62:413–4. 11037788 10.4269/ajtmh.2000.62.413

[24] Sardelis MR, Turell MJ, Dohm DJ, O'Guinn ML. Vector competence of selected North American *Culex* and *Coquillettidia* mosquitoes for West Nile virus. Emerg Infect Dis. 2001;7:1018–22. 10.3201/eid0706.010617 11747732 PMC2631924

[25] Godsey MS, Nasci R, Savage HM, Aspen S, King R, Powers A, Entomologic investigations during an outbreak of West Nile disease in southeastern Louisiana, 2002. Emerg Infect Dis. 2005;11. In press. 16229769 10.3201/eid1109.040443 PMC3310600

[26] Carpenter SJ, LaCasse WJ. Mosquitoes of North America, north of Mexico. Berkeley (CA): University of California Press; 1955.

[27] Komar N, Langevin S, Hinten S, Nemeth N, Edwards E, Hettler D, Experimental infection of North American birds with the New York 1999 strain of West Nile virus. Emerg Infect Dis. 2003;9:311–22. 12643825 10.3201/eid0903.020628 PMC2958552

[28] Tait N. The effect of temperature on the immune response in cold-blooded vertebrates. Physiol Zool. 1969;42:29–35.

[29] Ambrosius H. Immunoglobulins and antibody production in reptiles. In: Marchalonis J, editor. Comparative immunology. Oxford (UK): Blackwell Scientific; 1976:298–334.

[30] Cone R, Marchalonis JJ. Cellular and humoral aspects of the influence of environmental temperature on the immune resopnse of poikilothermic vertebrates. J Immunol. 1972;108:952–7. 5063311

[31] Cooper EL, Klempau AE, Zapata AG. Reptilian immunity. In: Gans C, editor. Biology of the reptilia. Chicago: University of Chicago Press; 1988. p. 298–352.

[32] Centers for Disease Control and Prevention. Possible West Nile virus transmission to an infant through breast feeding—Michigan, 2002. MMWR Morb Mortal Wkly Rep. 2002;51:877–8. 12375687

[33] Iwamoto M. Transmission of West Nile virus from an organ donor to four transplant recipients. N Engl J Med. 2003;348:2196–203. 10.1056/NEJMoa022987 12773646

[34] Pealer LN, Marfin AA, Petersen LR, Lanciotti RS, Page PL, Stramer SL, Transmission of West Nile virus through blood transfusion—United States, 2002. N Engl J Med. 2003;349:1236–45. 10.1056/NEJMoa030969 14500806

[35] Odelola HA, Oduye OO. West Nile virus infection of adult mice by oral route. Arch Virol. 1977;54:251–3. 10.1007/BF01314791 889447

[36] McLean RG, Ubico SR, Bourne D, Komar N. West Nile virus in livestock and wildlife. Curr Top Microbiol Immunol. 2002;267:271–308. 10.1007/978-3-642-59403-8_14 12082994

